# Musical Expertise and Second Language Learning

**DOI:** 10.3390/brainsci3020923

**Published:** 2013-06-06

**Authors:** Julie Chobert, Mireille Besson

**Affiliations:** 1 Laboratoire de Neurosciences Cognitives, CNRS—Aix-Marseille Université, 3 place Victor Hugo, 13331 Marseille Cedex 3, France; 2 Cuban Neuroscience Center, Ave 25 15202, Esq 158, Cubanacan, Playa, La Havane 11600, Cuba; E-Mail: mireille.besson@univ-amu.fr

**Keywords:** musical expertise, speech perception, speech production, phonetic contrasts, musicians, non-musicians, second language acquisition

## Abstract

Increasing evidence suggests that musical expertise influences brain organization and brain functions. Moreover, results at the behavioral and neurophysiological levels reveal that musical expertise positively influences several aspects of speech processing, from auditory perception to speech production. In this review, we focus on the main results of the literature that led to the idea that musical expertise may benefit second language acquisition. We discuss several interpretations that may account for the influence of musical expertise on speech processing in native and foreign languages, and we propose new directions for future research.

## 1. Introduction

Learning a second language (L2) is a real challenge. Multiple factors, including linguistic and extra-linguistic factors, are known to influence the acquisition of a second language and, in particular, the acquisition of non-native phonemic contrasts (e.g., [1]). The linguistic background of the learners, including the amount of knowledge in the native language (L1) (e.g., [2]), the proximity between L1 and L2 phonetic inventory (e.g. [3,4]) and the starting age of learning (e.g., [5]), are considered as the most important factors that determine learning performance. Moreover, extra-linguistic factors, like motivation [6], working memory [7,8], attention control [9,10] and, most interestingly for our concerns, musical experience, have also been shown to influence the perception and production of sounds in a foreign language (e.g., [11]). 

Music and speech share interesting similarities (for reviews, see [12,13,14]). Music and speech are complex auditory signals based on the same acoustic parameters: frequency, duration, intensity and timber. They comprise several levels of organization: morphology, phonology, semantics, syntax and pragmatics in language and rhythm, melody and harmony in music. Moreover, perceiving and producing music and speech require attention, memory and sensorimotor abilities. Finally, there is growing evidence that music and language share neural resources for processing prosody (e.g., [13,14,15]), syntax [16,17,18,19] and semantics [20]. Interestingly, musicians show improved abilities for speech processing (for recent reviews, see [12,21,22,23]). For instance, musical expertise positively influences different aspects of speech processing, such as prosodic modality, segmental and supra-segmental vocalic discriminations and the rhythmic structure of speech (see below). Importantly, such benefits have been reported for the native language, as well as for foreign languages (e.g., [24,25,26,27]), thereby suggesting that musical expertise may benefit second language acquisition. 

Several experiments have been conducted to test for this hypothesis. In this review, we first focus on studies that examined the relationship between musical expertise and the perception, identification and production of sound structure in native and foreign languages. We then consider an important aspect of learning foreign languages: the ability to segment a continuous speech flow into meaningful words or items. This ability, that also implies the implicit learning of syntactic rules based on statistical regularities between syllables, is enhanced by musical expertise and by musical training [28,29]. Finally, we discuss several interpretations that have been proposed in the literature to account for the positive influence of musical expertise on the processing of native and foreign linguistic sounds.

## 2. Sound Perception and Production in Native and Foreign Languages

Speech is a complex and temporally varying signal comprising different acoustic and linguistic properties that are necessary for understanding the intended message and for responding correctly. Here, we focus on two of the most studied acoustic parameters, frequency and duration, that define two perceptual attributes of sounds, pitch and duration. Pitch and duration contribute both to the melodic and rhythmic aspects of music and to the linguistic functions of speech. 

To recognize a spoken word, be it in the native language or in L2, the listener needs to analyze the acoustic and phonetic information contained in continuous speech. Language structure comprises two kinds of phonetic information: segmental and supra-segmental. Segmental information refers to the acoustic properties of speech that differentiate phonemes (consonant and vowel variations) used to convey differences between words. For instance, “bag” and “gag” differ from each other by one consonant that changes both the phoneme and the meaning of the word. Consonants and vowels are defined by phonetic parameters, like the place of articulation, voice onset time (VOT) and second formant transition (F2 transition) for consonants, as well as first and second formants (F1 and F2) for vowels. Supra-segmental information is concerned with the acoustic properties of more than one segment, such as intonation contours, stress patterns or prosody. Supra-segmental information also includes pitch information, as in tone languages, such as Mandarin Chinese, Cantonese, Thai and most African languages, in which pitch variations are linguistically relevant and determine the meaning of words (e.g., [30]). In Mandarin Chinese, for instance, there are four contrastive tones that change the meaning of the words (“ma” for instance): Tone 1 is high-level (ma (1) means “mother”), Tone 2 is high-rising (ma (2) means “hemp”), Tone 3 is low-dipping (ma (3) means “horse”) and Tone 4 is high-falling (ma (4) means “scold”). By contrast, quantity languages use variations of duration as supra-segmental cues. For instance, in Finnish, Hungarian or Japanese, vowel and/or consonant durations may change the meaning of the word (e.g., in Finnish “Tuli” means “fire” and “Tuuli” means “wind”).

The effect of musical expertise on pitch and duration perception in music and speech has been extensively studied in the literature, and results clearly reveal that musical expertise confers several linguistically relevant advantages (for recent reviews, see [12,21,22,23,31]. We focus on the experiments that tested for the effects of musical expertise on the perception and/or the production of supra-segmental and segmental cues varying in frequency and duration.

### 2.1. Perception of Frequency Cues

In a series of experiments, Besson and collaborators [24,32,33,34] investigated the effect of musical expertise on the processing of pitch variations in music and speech for native and foreign languages always using the same protocol. The design included musical and linguistic phrases that were ended with a congruous note/word for the one half and, for the other half, with a parametric manipulation of pitch: the final note was increased by 1/5 or 1/2 of a tone and the F0 contour of the final words was increased by 35% or 120% (supra-segmental changes), so that pitch variations were larger (easy to detect) or subtle (difficult to detect). In the first experiment, they compared musician and non-musician French adults [32]. Results revealed a lower percentage of errors to subtle pitch violations in musicians than in non-musicians not only in music, but also in their native language. Analysis of the event related potentials (ERPs) showed that this behavioral advantage was associated with a larger positivity (of the P3 family) to subtle pitch variations in both music and speech, but only in musicians. Similar results were reported in French children with four years of musical practice [33] and in a longitudinal study with non-musician Portuguese children musically trained for six months and presented with the same pitch manipulations as described above, but in spoken Portuguese sentences [34]. Taken together, these results clearly demonstrate enhanced pitch processing in both music and native speech processing, due to musical expertise. 

Turning to foreign languages, follow-up studies demonstrated that French adult musicians also perceived subtle pitch changes in Portuguese, a language that they did not understand, better than French non-musicians [24]. Moreover, the onset latency of the associated late positivity was 300 ms earlier in musicians than in non-musicians. Thus, these results also demonstrate the positive influence of musical expertise on the processing of prosodic modality in a foreign language.

In recent experiments, Jäncke and collaborators have investigated the influence of musical expertise on the perception of segmental contrasts in the native language. Interestingly, they found larger electrophysiological responses to voiced and unvoiced consonant in musicians than in non-musicians together with no between-group differences in behavior [35]. Moreover, using functional magnetic resonance imaging (fMRI), Elmer and collaborators [36] reported enhanced phonetic categorization, together with higher left planum temporale activation, in musicians compared to non-musicians. 

Turning to foreign languages, several experiments aimed at examining the influence of musical expertise on the discrimination of supra-segmental cues, such as non-native lexical tones [25,37,38,39,40,41,42]. At the behavioral level, Delogu and collaborators [38,39] asked Italian speakers, unfamiliar with tone languages, to perform a same-different task with sequences of monosyllabic Mandarin words. In both adults and children, results showed that melodic abilities and musical expertise enhanced the discrimination of lexical tones. However, the discrimination of segmental variations, such as consonant or vowel changes within a word, was not different between the two groups. Lee and Hung [41] also reported that English musicians were more accurate than non-musicians to identify intact syllables among syllables produced on four Mandarin tones that were either intact or modified in pitch height or pitch contour. 

At the brain level, results revealed how plasticity induced by musical expertise influenced lexical tone processing. Wong *et al.* [42] recorded the brainstem frequency following response (FFR) to Mandarin tone contour patterns in English amateur musicians and non-musicians, who were unfamiliar with tone languages. They reported higher quality of linguistic pitch encoding in the auditory brainstem responses of musicians compared to non-musicians, thereby suggesting that extensive experience with pitch information in musical context influences linguistic lexical-tone encoding. Moreover, very recently, Chandrasekaran and Kraus [43] demonstrated the relationship between the efficiency of inferior colliculus pitch representations (assessed by fMRI-adaptation) and the quality of neural pitch pattern representations (assessed by auditory brainstem recordings), this latter being known to be better in musicians than in non-musicians [42]. 

Recording event-related brain potentials (ERPs), Marie *et al.* [25] tested for the effect of musical expertise on the discrimination of tonal (supra-segmental) and segmental (consonant, vowel) variations in Mandarin Chinese in French musicians and non-musicians, unfamiliar with tone languages. Participants were auditorily presented with two sequences of four Mandarin Chinese monosyllabic words that were the same or different at the tonal level (e.g., *pà-kào-ná-gǎi vs. pà-kào-ná-gaì*) or at the segmental level (e.g., *bǎng-káo-mèn-bán vs. bǎng-káo-mèn-zán*). Musicians detected both tonal and segmental variations more accurately than non-musicians. Moreover, analysis of the ERPs revealed that tone variations were categorized faster by musicians than by non-musicians, as reflected by shorter latency N2/N3 components (see, also, [34,44]). Finally, the decision that tone and/or segmental variations were different was associated with larger P3b components [45,46] in musicians than in non-musicians. Thus, musical expertise was shown to improve the perception, as well as the categorization of segmental and supra-segmental linguistic contrasts in a foreign language. 

Taken together, studies of lexical tone perception by non-native listeners tend to show that listeners with a musical background discriminated and/or identified non-native lexical tones better than listeners without a musical background. Results also reveal more reliable encoding of linguistic pitch patterns at the subcortical level and enhanced discrimination and decision-related ERP components at the cortical level in musicians compared to non-musicians. 

### 2.2. Perception of Duration Cues

While most experiments included pitch variations in tone languages or in other speech sounds to examine pitch processing, fewer studies have examined the effect of musical expertise on the processing of duration. Based upon previous results by Magne *et al.* [47], Marie *et al.* [48] compared vowel duration and metric processing in continuous, natural speech in French non-musicians and musicians. They used a specific time-stretching algorithm [49] to create an unexpected lengthening of the penultimate syllable, thereby disrupting the metric structure of French words without modifying their timbre or frequency. They also manipulated the meaning of the final word of the sentence to create congruous or incongruous sentences. Participants performed two different tasks in two different blocks. In the metric task, they focused attention on the metric structure of the final words to decide whether they were correctly pronounced or not. In the semantic task, they focused attention on the meaning of the sentence to decide whether the final word was semantically expected within the sentence context or not. Musicians outperformed non-musicians (as measured by the percentage of errors) in both tasks. Moreover, the P2 component elicited by syllable lengthening was larger in musicians than in non-musicians, independently of the task performed. This was taken to reflect enhanced perceptual processing with enhanced musical expertise. Moreover, whereas P600 components were elicited in both tasks in musicians, they were only found in the metric task for non-musicians. Thus, musicians seem sensitive to the metric structure of words, independently of the direction of attention, that is, even if this information is not task-relevant. By contrast, the N400 effect was not different between the two groups, thereby showing no difference in semantic processing. 

While the Marie *et al.* [48] experiment was conducted in the native language of the listeners, Sadakata and Sekiyama [50] recently tested the hypothesis that musicians also outperformed non-musicians in processing supra-segmental duration variations in a foreign language. To this aim, they compared how Dutch and Japanese musicians and non-musicians process moraic features in Japanese. The mora is defined as a perceptual temporal unit and is used by Japanese listeners to segment speech signals [51,52]. For example, based on duration cues, a Japanese native listener will segment “hakkaku” into ha-Q-ka-ku (four morae), whereas a non-native listener will segment it into ha-ka-ku (three morae). They also tested participant’s perception of segmental vowel variations in Dutch. Vowels are mainly determined by combinations of formants, and categorical boundaries between Dutch and Japanese vowels do not overlap. They used the Dutch vowel u/Y/, which is between the Dutch vowels e/ε/ and oe/u/ and very close to the Japanese vowels e/e/ and u/u/, so that Japanese natives would encounter difficulties developing a new category for this Dutch vowel (e.g., [53]). 

The authors examined the categorical perception of both supra-segmental morae and segmental vowels variations by using both discrimination and identification tests. Whereas discrimination assesses the ability to compare acoustical cues without any knowledge of the target sounds, identification requires matching the characteristics of an incoming sound with pre-established category representations. Results of the same/different task with pairs of Japanese (e.g., kanyo-kannyo) and Dutch words (e.g., kuch-kech), differing in morae or vowel, respectively, showed that musicians, Dutch and Japanese, outperformed non-musicians in the discrimination of supra-segmental and segmental variations in their own language, as well as in the foreign language. Moreover, after learning these two categories, identification performance of moraic feature (in stop Japanese contrast) was higher in musicians (Japanese and Dutch) than in non-musicians. 

In sum, these results show that musical expertise enhanced the perception of the timing structure of speech both in native [48] and in foreign languages [50]. The Sadakata and Sekiyama [50] results are important, because they demonstrate that musical expertise not only influences the early stages of speech processing (perception and discrimination), but also categorical perception. In line with previous results of Gottfried and Riester [40] showing that English musicians unfamiliar with tone languages identified the four Mandarin tones better than non-musicians, these results raise the possibility that musical expertise enhances the ability to build reliable abstract phonological representations (e.g., [11,35]).

These results are also in line with those reported in children by Chobert *et al.* [54]. Musician children (*i.e.*, children on their way toward musicianship with an average of four years of musical training) were more sensitive (larger mismatch negativity (MMNs), lower error rate and shorter Reaction Times (RTs) than non-musician children (*i.e.*, who have not received musical training, apart from compulsory school education) to syllabic duration (a supra-segmental feature). Moreover, musician children were also more sensitive than non-musician children to small differences in voice onset time (VOT) that do not exist in their native language (larger MMNs and shorter RTs for large than for small VOT deviants). VOT is a fast temporal cue that allows differentiation of “ba” from “pa”, for instance, and that plays an important role in the development of phonological representations. By contrast, the MMNs and RTs recorded from non-musician children were equally sensitive to small and large differences in VOT (MMN and RTs were not significantly different for large and small deviants). In line with previous results by Phillips *et al.* [55] with non-musician adults, this was taken to indicate that non-musician children process all changes (whether large or small) as across-phonemic category changes [54]. 

Taken together, these results show that musicianship facilitates the learning of non-native supra-segmental and segmental contrasts defined by acoustical features (e.g., pitch and duration) and improves categorical perception. It may be that musical expertise refines the auditory perceptive system (bottom-up facilitation), but it may also be that years of intensive musical practice exert top-down facilitatory influences on auditory processing (e.g., [12,21,56]). These alternative interpretations are discussed in more detail in the final section.

### 2.3. Perception/Production Relationship

Turning to different aspects of speech processing, Slevc and Miyake [11] examined the relationship between musical and L2 abilities in four domains: phonology perception, phonology pronunciation, syntax and lexical knowledge. They tested 50 Japanese adults immersed in their L2 (English) after the age of 11 and controlled several factors, like the age of first L2 exposure, working memory and level of L2 use. Results of correlation analyses showed that musical abilities are predictive of phonological abilities (perception and production of the English /r/-/l/ contrast), but not of syntactic and lexical abilities. Investigations of the perception/production relationship in non-native languages are centered on the issue of whether performance in one domain influences the other domain. The Speech Learning Model postulates that production accuracy of non-native sounds is correlated with their perception [4], and several studies with bilinguals revealed significant correlations between perception and production of L2 segmental contrasts (e.g., [57]). By showing that musical expertise not only influenced the perception, but also the production of new phonological contrasts, these results are therefore in line with the Speech Learning Model.

Further evidence was provided by Tervaniemi and collaborators [23,27]. They investigated the relationship between musical aptitude and L2 phonemic discrimination and pronunciation skills in two studies with children and with adults. Musical aptitudes (as measured by the Seashore musicality test), language pronunciation (word repetition after a native speaker's model) and phonemic and chord discrimination tests (discrimination of phonemic dissimilarities between English and Finnish and between major chord and deviant chord) were assessed in 40 Finnish children (10 to 12 years old). In the pronunciation test, children were asked to repeat words containing phonemes that have no direct equivalent in Finnish (e.g., “television”, “measure” or “Asia”, which contain the sibilant /s/). Based on their level of performance at the English pronunciation test, children were divided into two groups. Results showed that children with advanced English pronunciation abilities had better musical skills than those who showed less accurate English pronunciation skills [26]. Moreover, Milovanov *et al.* [27] found the same pattern of results in Finnish young adults: participants with higher musical aptitudes were able to pronounce English better than participants with lower musical aptitudes. According to the authors, the positive correlation between general musical aptitude and level of performance in the English pronunciation test suggests an interconnection between musical aptitude and foreign language skills. 

Turning to lexical tone production, Gottfried *et al.* [58] showed that musicians (Native American English speakers) outperformed non-musicians to identify and produce the four phonemic tones of Mandarin. Gottfried and Ouyang [59] also reported that musicians pronounce Tone 4 (high falling) better than non-musicians. Acoustical analyses of the speech signal revealed a significant decrease in F0 from initial to final portions of the syllable in musicians’ T4 production, as typically found in native speakers, but not in non-musicians, demonstrating a positive influence of musical expertise on the phono-articulatory loop. This interpretation suggests that musical expertise may exert an influence on the dorsal pathway of speech processing described by Hickok and Poeppel [60] (see below). 

## 3. Language Segmentation

Together with the acquisition of L2 phonetic inventory, another major difficulty encountered by L2 learners is the ability to segment speech into separate words. Because word boundaries are not always marked by acoustic cues (pauses or stresses), the listener of a foreign language often perceives it as a continuous speech flow. Statistical learning has been proposed as centrally connected to language acquisition and development [61]. Typically, “syllables that are part of the same word tend to follow one another predictably, whereas syllables that span word boundaries do not” [62]. For instance, in “pretty baby”, the probability that “pre” is followed by “ty” (pretty) is higher than the probability that “pre” is followed by “ba”. The importance of transitional probabilities in speech segmentation has been demonstrated in adults, infants and neonates [61,63,64,65,66,67]. 

Statistical learning experiments are typically composed of a familiarization phase (learning) during which participants listen to a statistically structured continuous flow of artificial syllables, followed by a test, in which participants have to choose which of two items was part of the artificial language (the other item was built with similar syllables, but was not part of the language). Results of several experiments using both linguistic and non-linguistic sounds have shown that participants are able to segment the continuous stream by only using transitional probabilities (e.g., [68,69]). Moreover, sung language facilitates word segmentation compared to spoken language [70]. 

Recently, Francois and Schön [28] used a sung artificial language to test for the effect of musical expertise in adults on both melodic and word segmentation. The artificial language was constructed with 11 syllables combined into five tri-syllabic sung words (gimysy, mimosi, pogysi, pymiso and sipygy) with each syllable always associated with the same tone. Transitional probability within a word ranged between 0.5 and 1.0, whereas transitional probabilities across words ranged between 0.1 and 0.5. Participants passively listened to the sung artificial language and were tested with a two-alternative forced choice, with pairs of spoken words and melodies. While behavioral results did not reveal a clear-cut effect of musical expertise, ERP data showed larger N400-like components in musicians than in non-musicians in both the language and music tests. More recently, François *et al.* [29] conducted a longitudinal study over two school-years with 8–10-year-old non-musician children. Before training (T0), children were tested in two sessions. The first one included standard neuropsychological tests (WISC IV, [71]; Raven matrices, [72]), attentional tests (NEPSY, [73]) and speech assessments (ODEDYS, [74]). During the second session, EEG was recorded, and children were told to passively listen to the artificial sung language. The artificial language was adapted for children with nine syllables combined into four tri-syllabic words (gimysy, pogysi, pymiso, sipygy), each associated with a distinct tone. Based on children’s scores on the tests described above (T0), children were pseudo-randomly assigned to musical training or to painting training (control group), so as to ensure that there were no prior-to-training differences between groups. All children were tested again after approximately one year (T1) and again after approximately two years (T2) following the exact same procedure at T0. Both behavioral and electrophysiological measures showed a greater improvement in speech segmentation after musical training than after painting training. In sum, both musical expertise (in adults) and musical training (in children) improved speech segmentation of an artificial language, possibly because musicians built more reliable representations of both musical and linguistic structures during the learning phase. Importantly, the longitudinal approach allowed demonstration that the observed facilitation of speech segmentation more likely results from musical training than from genetic pre-dispositions for music (e.g., [34,75,76]). 

For methodological reasons, statistical learning experiments typically used an artificial language to control for the acoustic cues contained in the speech flow and that may serve as learning cues. However, what happens with natural language? Pelluchi, Hay and Saffran [77] conducted a statistical learning experiment with eight-month English infants listening to natural Italian stimuli. They demonstrated that after passive learning, infants were able to discriminate Italian items that belonged to the stream from new Italian items. These results provide evidence that infants used transitional probabilities for the segmentation of new words in a foreign language. An interesting perspective would be to determine whether the segmentation of a natural foreign language is also facilitated in musicians compared to non-musicians, children and adults. 

## 4. Interpretations and Future Research Directions

Several interpretations have been proposed to explain the facilitation of musical expertise on the perception and production of sounds in native and foreign languages. At the neuropsychological level, Patel [13] argued that processing the acoustic characteristics of music and speech relies on common processes. More specifically, the OPERA hypothesis [78] relies on the idea that the plasticity induced by musical practice occurs with the conjunction of five essential conditions: (1) overlap, of the brain regions that process acoustic cues in music and speech sounds, (2) precision, higher in terms of demand for musical training than for speech, (3) emotion, positive with musical activity, (4) repetition of musical activity, (5) attention, focus and engage with musical practice. In line with the shared resources hypothesis, results have shown that musical expertise is closely related to pitch awareness and phonological awareness [79]. Moreover, unvoiced stimuli, whether speech or non-speech, are processed differently by musicians and non-musicians [35]. 

Musical practice requires sustained attention control and memory. Several authors have pointed to the importance of attention in L2 learning success [9,10], and results have shown enhanced auditory attention (e.g., [56,80]) in musicians compared to non-musicians (for reviews, see [12,21,22]). Moreover, verbal memory is also strongly correlated with L2 vocabulary knowledge [81,82], L2 grammar (e.g., [83]) and L2 pronunciation [84] and, thereby, plays a crucial role in L2 learning. For instance, Kormos and Sáfár [85] found that working memory (assessed by the backward digit-span test) correlated both with measures of L2 ability (reading, speaking and listening) and with L2 vocabulary knowledge. Importantly, results also revealed improved working memory in musicians compared to non-musicians (e.g., [86,87,88,89,90,91]). Moreover, positive correlations were found between the duration of musical training and verbal working memory [92,93]. 

At the brain level, some brain imaging studies also showed larger activation of the working memory network in musical tasks in musicians than in non-musicians (e.g., [94,95,96]), and several results revealed that common brain regions are activated during verbal and music short-term memory tasks [97,98,99,100,101,102,103]. Enhancement of cognitive skills, such as attention and working memory with musical practice, is likely to facilitate L2 learning in musicians compared to non-musicians. 

Besson *et al.* [12] proposed that transfer of training effects may also facilitate specific aspects of speech processing, such as segmental and supra-segmental contrasts and prosodic processing. In line with this interpretation, available results suggest that musical expertise not only shape the activity of brain structures that are necessary for processing acoustic cues in speech, such as the brainstem, primary auditory cortex and supra-temporal gyrus, but may also influence the activity of other brain regions that are more specifically involved in phonological processing, such as the superior temporal sulcus [60,104] and the inferior frontal gyrus [105], regions that are known to be implicated in the learning of new speech contrasts [106]. According to the authors, the degree of learning success is related to the efficiency of the activation in frontal speech regions and of the deactivation in the temporal speech regions. Interestingly, Seppänen *et al.* [107,108] examined learning function in musicians and non-musicians in four consecutive oddball blocks with tones and showed larger decreasing N1, P2 and P3a/b source activation in musicians compared to non-musicians. They interpreted this result as an enhanced fast learning capacity in the auditory system to extract sounds features (N1 and P2) and as larger changes in attentional skills in musicians than in non-musicians (P3a/b).

Other interpretations are inspired from the Hickok and Poeppel dual route model of speech processing [60]. In this model, speech acoustic information is first processed in the superior temporal gyrus (STG) and then compared with a phonological representation in the superior temporal sulcus (STS). After these first stages, language processing is divided into two pathways: the dorsal pathway plays the role of a sensorimotor interface, allowing the mapping of phonological speech representations into articulatory representations. The ventral pathway, considered as a lexical conceptual interface, controls the mapping of phonological representations into lexical conceptual information. 

Based on this model, enhanced L2 pronunciation and speech segmentation in musicians compared to non-musicians may be explained by differences in the functioning of the brainstem and primary auditory cortex that lead to a reorganization of neurons along the auditory dorsal pathway (sensorimotor interface). It may also be that musicians develop more efficient connections in the dorsal pathway than non-musicians (e.g., [109,110]) and that the functional connectivity between the perceptive and sensorimotor systems is improved [111]. 

While the results reviewed above clearly show that musical expertise positively influences some aspects of L2 learning, such as the perception and production of new phonetic contrasts, more work is required to demonstrate that musical expertise facilitates the different processes involved in second language acquisition. For instance, results at the subcortical level clearly showed enhanced encoding of supra-segmental lexical tone contrasts in a foreign language [42]. However, to our knowledge, no study has yet examined the effect of musical expertise on the encoding of syllables that differ from the native language inventory by segmental variations (VOT, place of articulation, formants). Such studies would help determine if musical expertise also influences the subcortical encoding of very fine variations, such as the length of VOT or the F2 slope. 

Moreover, results at the cortical level also revealed better perception, discrimination and categorization of tones and L2 speech sounds in musicians compared to non-musicians (e.g., [24,25,42,50,112]). However, it would be of interest to further examine the influence of musical expertise on segmental speech variations in L2 or the perception of syllabic duration in quantity language. Marie *et al.* [25] examined the discrimination of segmental variations (consonant and vowels) in Mandarin by French musicians and non-musicians, and Chobert *et al.* [54] examined the preattentive processing of VOT contrasts that exist and do not exist in French with French children. Even if the influence of musical expertise on the perception of other important phonological contrasts, such as place of articulation, manner or formants still need to be examined. 

Maybe most importantly, second language acquisition requires learning new sound to meaning associations. An important direction for future research is, therefore, to determine whether musical expertise or musical training can facilitate the learning of such associations. A previous study by Wong and Perrachione [113] is very revealing in this respect. Adults native English speakers were asked to learn to associate an image of an object with English pseudowords superimposed on non-native pitch patterns (tones). Although musicianship was not manipulated in this study, results revealed that seven out of the nine successful learners were amateur musicians. Moreover, very recently, Chandrasekaran *et al.* [43] were able to demonstrate clear correlations between the efficiency (measured using an fMRI-adaptation paradigm), the faithfulness (measured with FFRs) of pitch representations in the inferior colliculus and the ability to learn pitch-to-word associations. Insofar as musicians encode both Mandarin tones and syllables characteristics in inferior colliculus with higher precision than non-musicians [42,114,115], it is tempting to speculate that musical expertise, by increasing sensitivity to the sound of a foreign language, might also facilitate sound-to-meaning association. 

Finally, it is important to keep in mind that correlation does not indicate causality and that the only way to test a causal link with musical training is to conduct a longitudinal study with non-musicians. To our knowledge, such studies have shown a positive effect of musical training on native language processing [29,34,116], but have not yet been conducted to test for the effect of musical training on foreign language processing.

## 5. Conclusion

Second language acquisition is a complex activity that requires numerous abilities, like precise encoding and perception of speech sounds, building solid representations, relevant word segmentation and sound-to-meaning association, appropriate pronunciation, as well as memory and attention abilities. In the present review, we described results demonstrating that musical expertise exerts a positive influence on several of these abilities. While more research is needed, the results reviewed above highlight the importance of musical expertise for perceiving and producing sounds in a foreign language. These results also open new perspectives for children with language-learning disorders who often show deficits in encoding speech sounds [117,118], in processing the temporal structure of speech sounds [119,120] and in the ability to construct solid phonological representations [121,122]. Moreover, children and adults with dyslexia often encounter increased difficulties in learning second languages, which may have life-long consequences (e.g., [123,124]). By shaping the auditory system and by improving auditory cognitive skills, musical training may help both children and adults to palliate some of their phonological deficits and facilitate second language acquisition. 
